# Updating changes in human gut microbial communities associated with *Clostridioides difficile* infection

**DOI:** 10.1080/19490976.2021.1966277

**Published:** 2021-09-05

**Authors:** Giovanny Herrera, Daniel Paredes-Sabja, Manuel Alfonso Patarroyo, Juan David Ramírez, Marina Muñoz

**Affiliations:** a Centro de Investigaciones en Microbiología y Biotecnología – UR (CIMBIUR), Facultad de Ciencias Naturales, Universidad Del Rosario, Bogotá, Colombia; b ANID – Millennium Science Initiative Program – Millennium Nucleus in the Biology of the Intestinal Microbiota, Santiago, Chile; c Department of Biology, Texas A&M University, College Station, TX, 77843, USA; d Molecular Biology and Immunology Department, Fundación Instituto de Inmunología de Colombia (FIDIC), Bogotá, Colombia; e Microbiology Department, Faculty of Medicine, Universidad Nacional de Colombia, Bogotá D.C. 111321, Colombia; f Health Sciences Division, Main Campus, Universidad Santo Tomás, Bogotá D.C. 110231, Colombia

**Keywords:** Gastrointestinal microbiota, *C. difficile*, microbial interaction, virome, irritable bowel syndrome

## Abstract

*Clostridioides difficile* is the causative agent of antibiotic-associated diarrhea, a worldwide public health problem. Different factors can promote the progression of *C. difficile* infection (CDI), mainly altered intestinal microbiota composition. Microbial species belonging to different domains (i.e., bacteria, archaea, eukaryotes, and even viruses) are synergistically and antagonistically associated with CDI. This review was aimed at updating changes regarding CDI-related human microbiota composition using recent data and an integral approach that included the different microorganism domains. The three domains of life contribute to intestinal microbiota homeostasis at different levels in which relationships among microorganisms could explain the wide range of clinical manifestations. A holistic understanding of intestinal ecosystem functioning will facilitate identifying new predictive factors for infection and developing better treatment and new diagnostic tools, thereby reducing this disease’s morbidity and mortality.

## Graphical abstract

**Figure:**
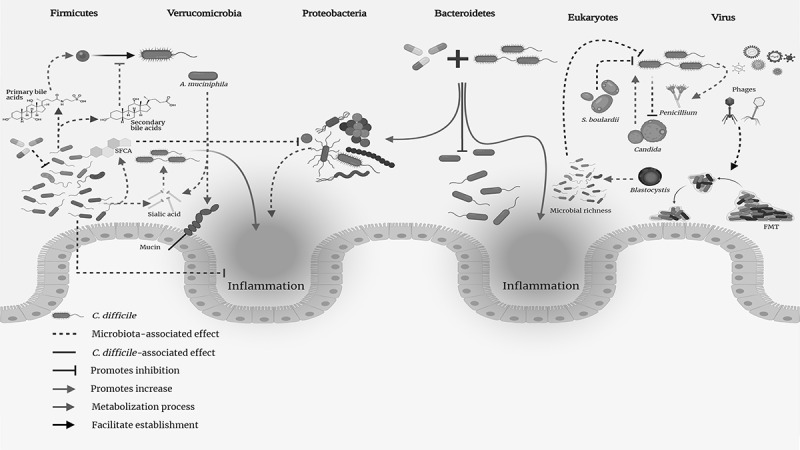

## Introduction

*Clostridioides difficile* (CD) infection (CDI) is a healthcare-associated infection, which has a substantial global impact, including antibiotic-associated diarrhea.^1,2^ This microorganism has a broad clinical spectrum, ranging from asymptomatic infections to complicated digestive tract illness that can lead to death.^1,2^ CDI represents a serious public health problem in developed countries; morbidity and mortality rates have increased during recent years, resulting in multimillion-dollar costs for health systems.^3,4^ Nevertheless, its impact on most developing countries remains unknown.

**Figure 1.**
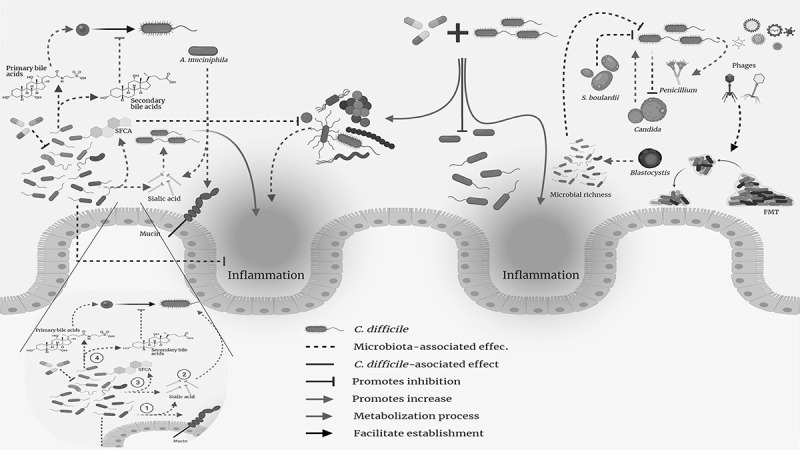
**Interaction between different bacterial phyla during *Clostridioides* *difficile* (CD) infection (CDI)**. Firmicutes. This phylum plays a primary role in defense against CDI and inhibiting intestinal inflammation as its members are mostly responsible for sialic acid metabolism to short-chain fatty acids (SCFAs) which prevent CD spore germination. SCFAs inhibit the growth of some pathogenic members from the Proteobacteria phylum. Verrucomicrobia. *Akkermansia muciniphila*’s role in this phylum is striking, given its involvement in colonocyte mucin degradation which increases sialic acid production, provides nutrient availability for the CD vegetative form and results in increased inflammation. Proteobacteria. CD decreases bacterial group abundance, thereby promoting the pathogenic bacteria growth and exacerbating CDI’s inflammatory symptoms. Bacteroidetes. Antibiotic use inhibits the growth of various members from this phylum, resulting in no increase in inflammation and the abundance of pathogenic bacterial phyla, such as Proteobacteria. Zoom panel: *Establishment of Clostridioides] difficile infection*. Under homeostasis, 1) intestinal bacteria metabolize carbohydrates from colonocyte glycoprotein membrane. 2) This results in sialic acid release which 3) is then degraded by commensal bacteria, generating SCFAs including butyrate (one of the main energy sources for colonocytes). 4) Primary bile acid conversion to secondary bile acid creates products preventing *C. difficile* spore passage to their vegetative form. However, these conditions are disturbed by antibiotic use – affecting commensal bacteria populations (in turn, protecting against intestinal inflammation), avoiding primary fatty acid conversion to secondary fatty acids and facilitating sialic acid availability which promotes *C. difficile* bacilli formation in any environment having little competition for energy sources

Patients undergoing antibiotic therapy in hospitals often suffer alterations in their intestinal microbiota as a result of treatment, thereby reducing the populations of beneficial microorganisms that compete for energy sources and metabolize primary bile acids into secondary bile acids, producing metabolites (i.e., taurocholic acid and glycolic acid) that inhibit CD growth (Figure 1).^3,5^ Antibiotic therapy is also known to alter populations of Archaea, such as *Methanobrevibacter* involved in bile acid metabolism.^6^

Other factors such as age and the use of some drugs can have an impact on microbiota, thereby facilitating CDI development (mainly concerning recurrent disease).^7^ However, intestinal homeostasis alterations are considered both a cause and consequence of CDI because the vegetative form of CD may promote indole production by pathogens, such as *Escherichia coli*, a bioactive molecule that inhibits protective gut microbiota growth and reconstitution during infection.^4^

The forgoing highlights the microbiota’s preponderant role regarding protection against CD colonization and the development of CDI itself; this has prompted various studies focused on describing the intestinal bacterial communities of individuals suffering CDI.^8,9^ Although studies have already highlighted intestinal microbiota components as effective tools for treating various diseases (i.e., CDI), extensive efforts are needed to understand its members’ functions regarding the intestinal ecosystem.^10^ An example would be the effectiveness of fecal microbiota transplantation (FMT) for restoring intestinal microbiota in patients suffering recurrent CDI, thereby leading to healing and a decrease in events associated with the disease, even surpassing the efficacy of antibiotic therapy schemes.^11,12^ However, limitations regarding the role of other microbiome constituents have not led to fully elucidating the key factors involved in this type of intervention’s success. The purpose of this review was thus to gather and discuss the main findings for a wide range of human gastrointestinal microbiota components and their relationship with CDI.

### Intestinal microbiome

The human gastrointestinal microbiome is a complex system of multiple microorganisms, their gene products and corresponding physiological functions.^13^ The microbiome includes bacteria, Archaea, viruses, and eukaryotic organisms constantly interacting with each other and with the target host.^14^ Microbiota composition is highly dynamic and depends on a host’s physical state, genotype, immune phenotype, and environmental factors, such as diet, antibiotic use, and lifestyle. Such environmental factors can adversely affect the intestinal ecosystem, their effects frequently being associated with increased susceptibility to infection and non-communicable diseases, such as obesity, metabolic syndromes (e.g., diabetes and cardiovascular disease), allergy, and other inflammatory diseases.^15^ Emerging evidence from recent studies has also established a two-way communication pathway linking the gastrointestinal tract and microbiota with the brain, suggesting that such microorganisms may play a role in neurological disorders as well as host perception, behavior, and emotional responses.^16,17^

Antibiotic use is associated with microbiota variations causing decreased diversity, an abundance of microbial communities, affecting the recovery of identical microbiota to that prior to long-term treatment;^18^ a differential effect has been observed regarding acute and recurrent CDI. It has been proposed that antibiotic treatment (or some other disturbance) significantly alters the composition of gut microbial communities,^19^ thereby having an impact on the balance of primary and secondary bile acids promoting CD colonization.^3^ Increased carbohydrate concentration (such as sialic acid) in the intestinal mucosa is a secondary effect of antibiotic therapy due to the disruption of carbohydrate-fermenting microbiota, which is exploited by CD during its proliferation.^20,21^

This has led to an increase in studies seeking to elucidate the relationship between the intestinal microbiota and CDI; most such studies have focused exclusively on bacterial populations.^3,4^ Although these populations are the primary components of microbial communities, other members play a determining role in maintaining intestinal homeostasis (i.e., Archaea, eukaryotes, and even viruses) and thus identifying other members represents a challenge for a complete description of gastrointestinal microbiome composition. *Tritrichomonas musculis* would be an example of the impact of protozoan species on this ecosystem; it has recently been revealed to be related to an increased intestinal immune response. Such immune responses (despite conferring resistance against colonization by certain bacterial species) have promoted inflammatory disease and tumor development in a murine model.^22^ *Blastocystis* subtype 7 has been seen to affect the microbiota by reducing the populations of beneficial bacteria, which could lead to an imbalance of the entire intestinal ecosystem.^23^

### Advances in microbiota research

Studying the intestinal microbiome has undergone numerous changes concerning data collection techniques and the tools for its analysis.^24,25^ Traditional and novel *in vitro* culture and techniques aimed at deciphering all microbiota components have recently led to the characterization of many bacteria in the gut.^26^ However, the challenges of culturing some fastidious microorganisms and difficulties regarding the recovery of other members of the intestinal ecosystem (such as viruses and eukaryotes) highlight the need for alternative molecular techniques for characterizing them.^27^ Next-generation sequencing and the advent of omics has led to the amount of microbiome studies increasing exponentially, thereby producing increasingly complex data regarding the members of this ecosystem and its homeostasis.

Single-marker amplicon-based sequencing is one of the most widely used methods for identifying microbiome components.^28^ This technique’s speed, ease, and reproducibility have made it a fundamental tool and an almost necessary first step when studying microbial ecosystems from intestinal and other sources; this has led to the discovery of multiple microbial communities inhabiting environments regarding which there was no prior evidence regarding their presence.^29^ This method is based on amplifying and sequencing marker genes’ (i.e., 16S-rRNA, 18S-rRNA and/or ITS) highly conserved regions among all groups. The presence of polymorphisms enables the identification and differentiation of the members belonging to a microbial community.^29^

Despite the multiple benefits of single-marker amplicon-based sequencing, it has been shown that this technique has some shortcomings hampering full understanding of all gut microbiota elements. The metagenomic approach has emerged as an alternative; it consists of the random amplification and sequencing of all genetic content in a sample.^27,29^ Metagenomics provides better taxonomic resolution and genomic information compared to single-marker techniques; it also facilitates the functional analysis and prediction of circulating genes.^30,31^ This technique’s cost can be prohibitive regarding the mass analysis of study populations and thus most studies involving this methodology have only used it on small population subgroups initially studied using just amplicon-based sequencing.^24,32^

### CDI and its impact on gastrointestinal microbiota

CD induces alterations in microbiota balance, ranging from asymptomatic infections to intestinal homeostasis imbalances, which can lead to serious symptoms and even death.^1,2,33^ Reduced diversity (i.e., different species in a sample) is one of the main alterations regarding intestinal microbiota; it is mainly caused by the decreased abundance of some groups of beneficial microorganisms and an increased abundance of pathogenic bacteria (Table 1).^34–36^

**Table 1.** Main findings from the study of *Clostridioides difficile*-associated bacterial microbiota

Year	Main findings	Study description	**Reference**
1982	Six genera inhibited *C. difficile* multiplication, *Lactobacillus* and group D Enterococci being the most antagonistic	*In vitro* study: 23 genera of fecal bacteria vs toxigenic *C. difficile* strains	^45^
1982	*Streptococcus* inhibited *C. difficile* growth	*In vitro* study of 7 *Streptococcus* strains vs 34 *C. difficile* strains	^46^
1988	Competition between unknown microorganisms and *C. difficile* by SFCA metabolism	*In vitro* continuous-flow culture model	^47^
1994	*C. difficile* failed to establish itself in the intestines of mice colonized with human fecal microorganisms; neither toxin A nor B were detected in these animals’ fecal pellets	Germ-free mice	^48^
1994	The combination of standard antibiotics and *S. boulardii* was shown to be an effective and safe therapy for patients suffering recurrent CDD; no *S. boulardii*-related benefit was demonstrated for those suffering an initial episode of CDD	A randomized placebo-controlled trial. 64 patients were enrolled having had an initial episode of CDD, and another 60 who had a history of at least one prior CDAD episode	^49^
2002	Altered composition of gut microbiota at species level in CDAD patients, characterized by greater diversity of facultative species, lactobacilli, and clostridia, but greatly reduced numbers of *Bacteroides, Prevotella and Bifidobacteria*	Identifying bacterial species isolated from healthy young adults and elderly subjects’ fecal samples and elderly patients suffering CDAD	^50^
2008	Recurrent CDAD patients had a highly variable bacterial community composition and decreased diversity	Stool samples from 10 individuals (7 CDAD and 3 controls)	^36^
2010	An increase in Firmicutes and Proteobacteria and a decrease of Bacteroidetes were observed	Nested case-control. 25 CDAD and 50 controls. 16S rRNA microarray	^32^
2012	Mice precolonized with a murine Lachnospiraceae isolate had significantly decreased *C. difficile* colonization, but not those colonized with *E. coli* while mice colonized with both *C. difficile* and *E. coli* died after 48 h (80% mortality reduction after 2 days in mice precolonized with *Lachnospiraceae* isolate)	Germ-free mice. Murine *Lachnospiraceae* and *E. coli* isolates were cultured from wild-type mice	^44^
2013	Decreased microbial diversity and species richness driven primarily by a paucity of phylotypes within the Firmicutes phylum. Normally abundant gut commensal organisms, including the Ruminococcaceae and Lachnospiraceae families and butyrate producing C2 to C4 anaerobic fermenters, were significantly depleted in CDI and CDN groups	Culture-independent high-density Roche 454 pyrosequencing was used to survey the distal gut microbiota for 39 individuals having CDI, 36 subjects suffering (CDN), and 40 healthy control subjects	^51^
2015	CDI patients and asymptomatic carriers had microbial richness and diversity compared to healthy subjects, accompanied by a paucity of phylum Bacteroidetes and Firmicutes and overabundance of Proteobacteria. Some normally commensal bacteria, especially butyrate producers, were significantly depleted in CDI patients and asymptomatic carriers	25 participants (8 CDI patients, (asymptomatic *C. difficile* carriers) and 9 healthy individuals)	^35^
2016	Increased butyrogenic bacteria in both CDI and non-CDI patients. Increased *Akkermansia* and *Penicillium* in CDI patients. Decreased *Bacteroides* population density	24 inpatients with diarrhea (12 CDI vs 12 controls)	^43^
2016	Lower microbial diversity in CDI patients. CDI was associated with a significant under-representation of gut commensals having putative protective functionalities, including *Bacteroides, Alistipes, Lachnospira* and *Barnesiella*, and over-representation of opportunistic pathogens	Three groups of hospitalized elderly patients (age ≥ 65) following standard diet including 25 CDI-positive (CDI group), 29 CDI-negative exposed to antibiotic treatment (AB+ group) and 30 CDI-negative subjects not on antibiotic treatment (AB− group)	^37^
2016	A review highlighting risk factors for developing CDI. CDI patients had increased Proteobacteria and decreased commensal bacteria: *Ruminococcaceae, Lachnospiraceae* or *Bifidobacterium longum*	Review	^7^
2016	Metabolomics profiling (highly responsive to changes in physiological conditions) has shown promise in differentiating subtle disease phenotypes having a nearly identical microbiome community structure, suggesting metabolite-based biomarkers may be an ideal diagnostic tool for identifying CDI patients	Review	^42^
2017	The authors identified *C. difficile* in 131 of 156 CDI index cases (1.78% average abundance) and 18 out of 211 healthy controls (0.008% average abundance). Consistent negative association with *C. scindens* and multiple *Blautia* species	High-resolution method for 16S rRNA sequence assignment to previously published gut microbiome studies of CDI and other patient populations	^52^
2018	Microbiota-dependent alteration in innate immune response early on during infection may explain poor outcome in aged hosts suffering CDI	*in vivo* mouse model	^53^
2018	Compared to IBD patients without CDI, IBD patients with CDI had more pronounced dysbiosis with higher levels of *Ruminococcus gnavus* and *Enterococcus* OTUs and lower levels of *Blautia* and *Dorea* OTUs	56 IBD patients, including 8 having flares with concomitant CDI, 24 flares without CDI, and 24 in remission; 24 healthy subjects	^54^
2018	Supplementing with anti-inflammatory butyrate-supporting commensal bacteria and prebiotics may support innate immune responses and minimize bacterial burden and negative effects during antibiotic treatment and exposure to CD	*in vivo* mouse model	^41^
2019	A reduced abundance of *Bacteroides* was associated with a poor CDI prognosis, severe diarrhea, and high recurrence incidence	57 patients suffering diarrhea from nosocomial and community-acquired CDI	^9^
2019	Several genera, such as *Phascolarctobacterium, Lachnospira, Butyricimonas, Catenibacterium, Paraprevotella, Odoribacter*, and *Anaerostipes*, were not detected in most CDI cases	79 tcdB positive patients and 20 controls	^38^
2020	Depletion of *Alistipes* and *Ruminococcus* species and reduced methionine biosynthesis were noted in *C. difficile* patients having undergone surgery	A prospective single-center study of 70 CD patients	^40^
2020	66 species inhibited *C. difficile*; species composition and blend size were important re inhibition	*C. difficile* coculture with 1,590 isolates from gut microbiota. 256 combinatorial community assemblies	^55^
2021	There was a significant association between *Blastocystis* and CDI	220 patients suffering diarrhea	^56^

SCFA: Short-chain fatty acids; CDD: *Clostridioides difficile*-associated disease; CDAD: *Clostridioides difficile*-associated diarrhea; CD: *Clostridioides difficile*; CDI: *Clostridioides difficile* infection; OUT: operational taxonomic units

The phylum Firmicutes is one of the groups having decreased abundance following CDI; bacterial families such as Ruminococcaceae and Lachnospiraceae belong to it (Table 1).^8,37,38^ Such bacteria are known for their role in butyrate production which is the preferred metabolic substrate for colonocytes; butyrate metabolism contributes to maintaining low oxygen levels, thereby suppressing pathogenic aerobic and facultative bacteria populations (Figure 1).^39^ Reduced *Faecalibacterium* and *Bifidobacterium* counts are apparent in these groups and such changes have been associated with intestinal anti-inflammatory effects, which would explain their depletion in CDI.^40–42^ Most individuals included in studies providing evidence of such alterations had been treated with antibiotics, probably leading to a reduction of these populations.^37,43^ However, Reeves *et al*.,^44^ have advanced an argument favoring these microorganisms’ role through experiments using germ-free mice colonized with a Lachnospiraceae murine isolate in the absence of *E. coli*; they observed partial restoration of resistance to CDI (Table 1). Such findings have highlighted these microorganisms’ preponderant role in CDI prevention.

Regarding the description of specific microorganism genera and their relationship with CDI, a finding in mice has indicated that CDI in a murine model was related to a decreased abundance of specific groups of microorganisms; this included *Clostridium scindens* (Table 1) a bile acid dehydroxylator acting as protector via the production of primary bile acid-derived metabolites, thereby making it a probiotic candidate for CDI treatment.^57^ Another study reported increased *Akkermansia muciniphila* abundance in CDI patients.^43^ This microorganism has frequently been associated with healthy intestinal microbiota, mainly in obesity studies.^58^ This finding has been associated with this microorganism’s ability to degrade mucin in the intestine’s mucous layer; its metabolites are used by CD as an energy source, thereby enabling it to survive in the environment (Figure 1).^8,43^

There is usually a decreased abundance of some butyrate-producing bacterial genera in CDI patients, including *Dorea* and *Blautia* spp. (Table 1); their reestablishment in intestinal microbiota can thus prevent CD spore germination through primary bile salt metabolism.^34,52,54,59^ These findings stress the fact that intestinal microbiome composition and its members’ functions must be evaluated according to the global scenario being studied. The influence of inter-individual variations regarding these microorganisms’ role and on the metabolic pathways in which their participation has been suggested must also be deciphered.

A similar situation has been observed in the phylum Bacteroidetes^32^ within which such genera as *Alistipes, Prevotella*, and *Bacteroides* are usually associated with intestinal mucosa inflammation (Table 1).^37,40,50,53^ Such reduction is usually accompanied by an increased abundance of members from the phylum Proteobacteria (Table 1) which are known for their role in disrupting intestinal homeostasis and mucosal inflammation. This results in exacerbating intestinal symptoms and leads to clinical complications that could eventually result in patient death (Figure 1).^35,51,60^

The aforementioned relationships between microbiota members denotes complex crosstalk systems within a competitive ecosystem (that could work in a bidirectional and dynamic manner) in which certain populations are constantly replaced by others. This would have an impact on these microbes’ functions and consequences concerning tissues and affect the intestine’s delicate balance. Intestinal regulation between inflammation and repair varies depending on the environment, nutrient availability, and their components.^61^ This is reflected in the effects that small changes in certain groups of microorganisms have on such balance; maintaining intestinal homeostasis thus represents a challenge for modern science.

### In search of the optimal microbial composition or restoration of intestinal homeostasis regarding CDI

CDI-related gastrointestinal microbiota studies have shed light on the affected components and their impact on microbiota balance; research has thus been focused on restoring gastrointestinal microbiota through FMT. The first studies were aimed at determining the microorganisms directly involved in protection against CDI to produce an adequate cocktail of microorganisms for restoring the balance of intestinal homeostasis. Some research has demonstrated that certain bacteria from the genera *Streptococcus, Lactobacillus*, and *Bacteroides* have inhibited CD growth, possibly through competitive effects on the monosaccharides released from mucin (Table 1).^45–47,62^ Evidence has emerged regarding the role of intestinal microbiota as a barrier against CDI and the impact that antibiotics and other medications have on its balance to promote the microorganism’s spore germination.^48,63^

Later, studies have focused on FMT from a healthy individual to one having CDI and its impact on microbiota restoration. One such study reported a dramatic change in the recipient’s microbiota composition 14 days after transplantation; similarity with donor microbiota was achieved, leading to symptom resolution.^64^ However, such interventions were unsuccessful in some patients; this led to further research regarding specific components and appropriate administration routes for this type of treatment. Different phyla and bacterial families were identified as potential CD biomarkers and antagonists.^6,43,55^

*Lactobacillus* and *Saccharomyces boulardii* administration has proved effective in CDI patients’ treatment and recovery in some studies.^49,65,66^ However, subsequent studies did not corroborate such findings and even suggested that *Lactobacillus* administration as a probiotic is contraindicated for critically ill patients because of the risk of fungemia.^56^ This finding led to the development of therapy guidelines indicating the types of patient for whom FMT can be suggested.^67–69^

The US Food and Drug Administration and the international consensus conference on stool banking for FMT in clinical practice determined the criteria that donor patients must meet, that is, the absence of sexually-transmitted infection, intestinal disorders, and other risk factors along with disorders and drug use that could alter intestinal microbiota. The absence of microorganisms, such as CD, common enteric pathogens, such as *E. coli, Salmonella, Shigella*, and *Vibrio*, and antibiotic-resistant bacteria, such as vancomycin-resistant *Enterococcus* and methicillin-resistant *Staphylococcus aureus* must be guaranteed. There had to be lack of some viruses (i.e., norovirus and rotavirus), helminths and protozoa (i.e., *Blastocystis, Dientamoeba, and Giardia*) and microsporidia.^68,69^

Despite such advances, identifying the right combination for restoring intestinal microbiota that can be administered to patients without causing side effects remains a challenge for the scientific community. Although several studies have suggested different FMT combinations, a recent meta-analysis has shown that a decisive aspect concerning this treatment’s effectiveness was related to its administration route and the amount used.^70^ The aforementioned findings suggest that other intestinal ecosystem components could be involved in FMT success or failure, preparing the way for new studies assessing previously unexplored intestinal microbiota components, such as viruses and eukaryotes.

### Archaea: small populations having a significant impact

Archaea are a large and diverse group of abundantly-distributed, single-celled, intestinal microorganisms; their variations in terms of abundance are related to geographical and ethnic factors.^71^ This domain’s role was poorly studied for some time because of its low abundance and such microbes were considered to have a low impact on microbiota homeostasis.^71^ The main members of this group account for less than 2% of intestinal microbiota methanogenic microorganisms, microbes, such as *Methanobrevibacter*, and halophilic ones including *Haloferax* and *Halococcus*.^72^

Heterogeneous roles such as gut microbiota have been associated with Archaea; their role as probiotics has been mentioned because they can metabolize intestinal products that can be harmful to health; the term ‘archaebiotics’ is consequently the subject of ongoing research.^72,73^ Hydrogen consumption is another role associated with this domain’s members; it could increase ATP availability generated by anaerobic bacteria, thereby creating an ideal setting for the growth of opportunistic populations and causing symptoms such as constipation.^72^ It is worth highlighting *M. smithii*’s protective role regarding inflammatory bowel disease (IBD) due to its ability to produce short-chain fatty acids (SCFAs) which could act as CDI-related protectors.^6^ An opposite effect has been observed for *Methanosphaera stadtmanae*, which is frequently associated with IBD.^61,74^

Despite this domain’s low abundance, it has a great impact on gut microbiome as shown by studies regarding obesity, muscle abscesses, pneumonia, and urinary tract infection.^71^ Research has shown that members such as *M. smithii* can play a protective role against CDI due to SCFA-associated mechanisms; the reduction in this Archaea’s relative abundance has been related to diarrheal symptomatology and CD proliferation.^75,76^ Inflammatory diseases associated with these populations’ imbalance suggests so-far-unknown mechanisms, so future in-depth studies must ascertain microbiota components in CDI and other diseases.

### Protists, helminths, and fungi: poorly explored territory?

Eukaryotic microorganisms residing in many vertebrate species’ gastrointestinal tracts also affect host health/disease events; however, characterizing such microbiome components has lagged behind that for bacteria.^77^ Some multicellular (i.e., helminths) and unicellular organisms (amoebae, some fungi, and certain protozoa) have been identified as members of the gastrointestinal microbiome. Many of these taxa have been investigated from a parasitological viewpoint for decades now, using microscopy and directed molecular approaches. It is thus considered that eukaryotic microbiota diversity in the human intestine has not yet been systematically investigated from a community perspective.^78^

The relationship between eukaryotic microorganisms and intestinal diseases in humans has been established; however, recent advances have led to understanding that not all eukaryotes in the intestinal tract should be considered parasites as many of them increase bacterial diversity and interact with the immune system to prevent pathogens’ intestinal colonization.^79^ Although, the pathogenic roles of eukaryotic species have been documented (i.e., *Ascaris lumbricoides, Entamoeba histolytica, Cryptosporidium* spp. and *Strongyloides stercoralis*), recent evidence has suggested that other eukaryotic microorganisms commonly inhabiting the gastrointestinal tract may play significant ecological roles in intestinal homeostasis (i.e., *Blastocystis* and *Dientamoeba*).^78–81^

*Blastocystis* represents a special case deserving more detailed study because of contradictory research results (Table 2). Evidence of its pathogenic role regarding human health is extensive, as is the effect that it can have on intestinal microbiota. Colonization by this protozoan has been associated with healthy microbiota because increased intestinal microbiota diversity has been observed, together with less abundance of Enterobacteriaceae in *Blastocystis*-positive patients.^87^ However, another study of irritable bowel syndrome (IBS) patients did not reveal differences regarding microbiota composition and diversity compared to that of healthy controls.^88^ Recent research involving school-aged children in Colombia indicated that *Blastocystis* was accompanied by decreased abundance of *Bacteroides* and increased relative abundance of *Faecalibacterium*, although change in intestinal microbiota composition was not statistically significant.^89^

**Table 2.** Microbiota changes caused by human protozoa

Protozoa	Bacterial group altered	Effect	Study type	**Ref.**
*Giardia lamblia*	Beneficial bacterial groups	Induces alterations aggravating *Giardia-*associated symptoms	*In vitro* cell culture and germ-free murine infection model	^82^
*Giardia lamblia*	*Clostridium, Lactobacillus* and *Bacteroides* in canines *Prevotella* and *Gammaproteobacteria*	Increased bacterial diversity and beneficial groups. Decreased potential pathogenic bacteria	Cross-sectional and data mining	^83^
*Entamoeba histolytica*	*Prevotella copri*	Increased bacterial group induced colitis	*In vivo*	^84,85^
*Entamoeba coli*	*Akkermansia*	Increased beneficial bacteria could have led to establishing healthy microbiota	Cross-sectional	^86^
*Blastocystis*	*Enterobacteriaceae*	Increased microbial diversity and lower abundance of potential pathogenic bacterial group	Cross-sectional	^87^
*Blastocystis*	-	No differences between *Blastocystis-*infected and control groups	Cross-sectional metataxonomic	^88^
*Blastocystis*	*Bacteroides* and *Faecalibacterium*	No statistically significant differences in microbiota composition	Cross-sectional	^89^
*Blastocystis*	*Bacteroides, Prevotella* and *Ruminococcus*	*Bacteroides-*driven enterotype could protect against *Blastocystis* infection	Metagenomic	^90^
*Blastocystis*	*Bifidobacterium* and *Lactobacillus*	*Blastocystis* subtype 7 could induce alterations in beneficial bacterial groups	*In vitro* and *in vivo*	^23^
*Blastocystis*	*C. difficile*	Co-infection with both microorganisms suggested alternative mechanisms for *Blastocystis* adaptation	Cross-sectional	^56^

Metagenomic studies have attempted to resolve such contradictory findings. Andersen *et al*., observed that people having *Bacteroides*-dominated microbiota were less prone to *Blastocystis* colonization than those whose microbiota was dominated by *Prevotella* and *Ruminococcus*.^90^ These findings did not delve into the *Blastocystis* subtype involved in such infection. Some subtypes have been associated with intestinal manifestations, such as subtype 7; Yason *et al*., described its impact on intestinal microbiota using *in vivo* and *in vitro* techniques. They observed negative effects on beneficial bacteria (i.e., *Bifidobacterium* and *Lactobacillus*) which could have led to microbiota dysbiosis, thereby facilitating the appearance of intestinal pathologies.^23^

**Figure 2.**
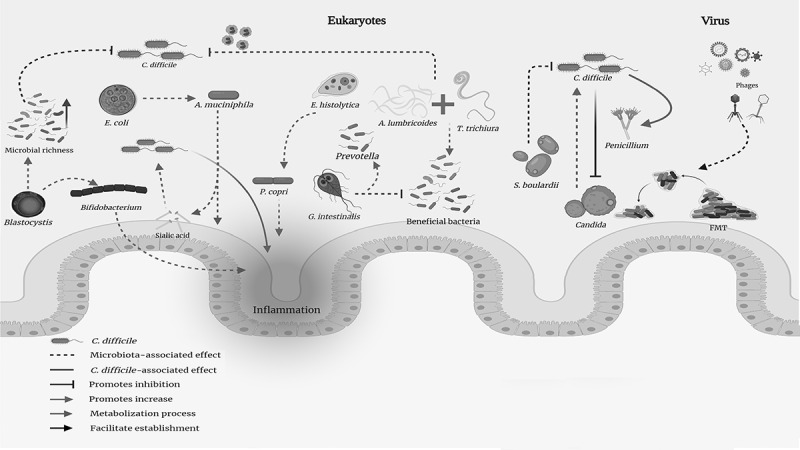
**Interaction between different members of the eukaryotic and viral microbiota during *Clostridioides* *difficile* (CD) infection**. *Blastocystis* has been associated with increased microbial richness, resulting in a state of protection against various intestinal diseases. It has also been associated with increased abundance of *Bifidobacterium*, a genus capable of triggering an increase in intestinal inflammatory activity. *Entamoeba coli* has been associated with an increased abundance of *Akkermansia muciniphila* and intestinal microbial structure comparable to that of healthy subjects. *E. histolytica* is associated with increased prevalence of *Prevotella copri* which is sometimes used as a biomarker for diarrheal disease in cases of amoebiasis and inflammatory bowel disease. *Giardia*. This protozoan has been associated with increased *Prevotella* prevalence and decreases in beneficial bacteria populations. Nematodes. Co-infection by *Ascaris lumbricoides* and *Trichuris trichiura* leads to increased abundance of beneficial bacteria; such change could lead to asymptomatic infection by other pathogens. Fungi. *Saccharomyces boulardii* has been associated with *in vivo* and *in vitro* CD growth inhibition. The opposite occurs with *Candida* (a genus that can enhance bacillus growth); however, it has also been shown that the bacillus inhibits the growth of different species of the fungus. *Penicillium* has been associated with increased CD, taking advantage of imbalance in microbiota caused by the bacillus. Virus. Although different viruses have been described as forming part of the intestinal microbiota (rotavirus, astrovirus, calicivirus, norovirus, hepatitis E virus, coronavirus, torovirus, and adenovirus predominating), their roles or intestinal interactions are unclear. Phages are associated with microbiota establishment after fecal microbiota transplantation

Vega *et al*., recently described *Blastocystis* in CDI patients, highlighting the adaptive mechanisms enabling this protozoan to survive in CD-related imbalance (Figure 2).^91^ Vega *et al*., (in other work) suggested that *Blastocystis* and CD co-occurrence could positively modulate intestinal microbiota, permitting increased beneficial bacteria abundance compared to patients without *Blastocystis*.^56^ There were no differences between groups regarding eukaryotic microbiota abundance. This highlights the need to explore this important microbiota component because its impact remains unknown.

The helminths (generally considered pathogenic) have contributed to increased intestinal microbiota diversity which tends to disappear after therapy aimed at their removal;^92^ as with protozoa, contradictory results have been found. A study of children in Ecuador co-infected with *Trichuris trichiura* and **Ascaris* lumbricoides* observed decreases in Firmicutes abundance and reduced bacterial diversity, which did not occur in children only infected by *T. trichiura*,^93^ denoting *A. lumbricoides* influence on intestinal microbiota modulation. Another study in Malaysia recorded increased bacterial diversity in samples from helminth-infected children and increased abundance of bacterial species belonging to the Paraprevotellaceae family in *T. trichiura*-infected individuals.^94^ A study of celiac disease patients assessed the impact of *Necator americanus* infection; increased bacterial richness was observed.^95^

Such relationships suggest that hosts and parasites do not exist in an isolated manner but that they interact via co-evolution, enabling the balanced, co-existence of countless microorganisms in a niche benefiting all members, including the host^79^ (i.e., a two-way relationship in which such interactions have a positive or negative impact on other members of the microbiota).^92^ Such relationships could consequently explain CDI patients’ clinical manifestations, since some eukaryotes’ positive modulation of the microbiota could protect against inflammation and diarrhea, creating a delicate balance resembling a healthy patient’s microbiota.^35^ It has been suggested recently that helminth infection could be a protective factor for CDI due to the type-2 immune response promoted in a host during such infection and eosinophil proliferation, which can reduce CD populations by still-unknown mechanisms.^96^ Host immune response represents an interesting field of study having profound pathophysiological implications and even new therapeutic options for CDI as occurs with other inflammatory diseases, such as Crohn’s disease and IBD where *Trichuris suis* use has been suggested as possible treatment.^97^

Intestinal mycobiome composition (microbiota fungal components) has been less extensively studied. The main mycobiome components have been identified as *Saccharomyces, Malassezia*, and *Candida* in a study involving a cohort of healthy patients; the role of *Candida* in CDI establishment and development, however, is not entirely clear because of conflicting results (Figure 2).^98–101^ Some studies have found a correlation between great *C. albicans* abundance and reduced FMT efficacy,^102^ while other research has recorded low *C. albicans* frequency in CD-colonized patients and described a probable protective role for *Candida* species overgrowth regarding CDI and its lethal effects.^103,104^

No evidence has been presented concerning the relationship of *Malassezia* with CDI; however, recent studies have suggested that intestinal conditions may promote its growth and colonization, which could lead to exacerbation of IBS symptoms.^105^ The protective effect of *Saccharomyces boulardii* against colitis caused by CD has been demonstrated (i.e., one of the most common probiotics isolated from fruits); this is mediated by immunoglobulin A production (Figure 2).^106,107^ Increased *Penicillium* abundance has been observed in CDI patients suggesting that intestinal fungal microbiota imbalance may contribute to CD (Figure 2).^55^ However, no recent evidence has supported the role of this or any other fungi regarding CDI development.

### The unexplored virome in CDI

The gut virome (defined as all viruses inhabiting the intestinal tract) consists of bacteriophages (phages) that infect bacteria, viruses that infect other cellular microorganisms (such as Archaea), eukaryotes (i.e., protozoa or human cells) and free viral particles as transients in food.^108^ Such viruses (including DNA and RNA viruses) have become increasingly important regarding the gastrointestinal tract because of their contribution to microbial ecology, meaning that their diversity, abundance, and function in intestinal microbiota must be compared.^109^

A limitation of virome sequencing concerns suitable methods for purifying and enriching all ranges of virus-like particles from stool samples. There are also genome-related limiting factors (size and composition, especially regarding RNA viruses); the small percentage within microbial communities could result in underestimating their participation in such ecosystems.^110^ These factors have led to standardizing sample processing methods, ascertaining evaluation of such particles’ true representativeness and their characterization.^111^

Despite limitations, some studies have led to improving the intestinal virome’s description; some representative groups have been identified, including double-stranded DNA and RNA viruses,^112–114^ that could have an impact on microbial communities’ modulation and consequent effects on host health. It is worth describing these particles’ dynamics.^115^

Few CDI studies have focused on clarifying the relationship between viral microbiota and CD. CDI patients’ intestinal virome was first characterized in 2018 in Asia (Table 1); a dysbiotic enteric virome was demonstrated in this study, mainly characterized by a decreased abundance of Microviridae family viruses.^116^ Further studies were aimed at determining modifications and impact on the virome after FMT was used for CDI treatment. Several studies have shown that an intestinal viral core is specific for each donor’s conditions despite modifications to the microbiota following FMT; this is characterized by a decrease in Caudovirales, though retaining a phage profile similar to that of unaltered individuals. This has suggested that this component contributes to the long-term establishment of donor microbiota (Table 1, Figure 2).^116–118^

The aforementioned research strikingly indicated that although phages are markers of inflammation, their abundance does not vary between donors and recipients. Future research must thus delve into the role played by these microbiota components. Phages’ beneficial role has also been observed regarding metabolism, motility regulation, and maintenance of the intestinal barrier against pathogens;^119–121^ regardless of such evidence, this topic has not been extensively studied. Some viruses’ ability to infect *Entamoeba* and *Giardia* (highly prevalent parasites worldwide) have been demonstrated recently;^122^ however, such findings’ impact must be clarified. This data supports the need to assess other intestinal ecosystem components’ roles and the resulting interactions between members of the different kingdoms and their health–disease-related implications.

### Interdomain complexities: a holistic view of intestinal ecosystem CDI

The intestinal ecosystem should not be viewed or analyzed as a sum of isolated components; rather, it should be understood as a complex network of interactions among its different elements. Established and speculated relationships between gastrointestinal microbiota members highlight the microbiome’s complexity (Figure 2). Concerning CDI, evidence has been presented regarding interactions among the domains inhabiting the intestinal ecosystem, including interdomain communication pathways mediated by signaling molecules, such as indoles;^123^ metabolites produced by some members of the microbiota promoting other members’ survival has been highlighted. SCFAs represent one relevant example based on evidence of their use by both bacteria and eukaryotes using them as energy supplies.^124^

This complex interaction fulfills energy needs/functions; intestinal microbiota members’ ability to modulate host immune response has also been shown, suggesting asymptomatic infections (as observed for some protozoa) or symptom exacerbation (as observed in IBS).^84,85,125^ The above is especially important as intestinal microbiota can maintain a delicate balance with the mucosa’s immune system by regulating antigen presentation, thereby enabling/ensuring the survival of many of this ecosystem’s inhabitants.^126,127^ This balance can be affected by many factors, such as parasites able to modulate the immune response thereby activating mechanisms (such as the inflammasome) ultimately exacerbating intestinal inflammatory symptoms due to commensals being recognized as foreign agents.^22^ This also occurs for microorganisms directly affecting the intestinal mucosa, resulting in the release of immune system cells and pro-inflammatory molecules interacting with usual microbiota members. They consequently become targets for an aberrant immune response.^128,129^

The gastrointestinal ecosystem’s complex relationships must be comprehensively explored for a better understanding of the findings. Antibiotic-associated diarrhea is a clear example of this as the fragmented study of a complex network of relationships and interactions does not provide a complete picture of the disease’s pathophysiology. This constitutes a challenge for future research aimed at covering as many components of the intestinal ecosystem as possible (microbiome, metabolome, and interactome) and replicate its conditions in the most reliable manner possible to ensure obtaining accurate results to improve the health of millions of people worldwide. This challenge implies understanding the imbalance in microbiota that occurs during CDI from many perspectives, including biotic components and immunological and molecular factors that may be involved in the disease. Future studies focusing on these factors should lead to complete understanding of the phenomenon.^82,83,86^

## Conclusions

Intestinal microbiota members’ effects on homeostasis and diseases are highly variable and even contradictory. Many difficulties related to studying microbiota in relation to CDI arise from the impossibility of controlling the confounding factors, along with the approach used for conducting these studies. Such approaches usually examine small groups of microbiota members of a complex and constantly changing ecosystem. The microbiota is increasingly presented as a complex ecological niche of constantly evolving interactions, which must be reconsidered regarding its study and analysis. New perspectives must enable a vision encompassing most, if not all, of the parts encompassing the intestinal microbiota.

Most microbiota–CDI research has been limited to examining the role of bacteria in relation to CDI establishment and development. Although much of the knowledge regarding the disease’s pathophysiology is derived from such studies, large gaps remain regarding a complete and multifactorial understanding of intestinal imbalance because of the role played in intestinal diseases by other ecosystem elements. Although their role was assumed to be practically nil, current evidence has indicated that they could be main actors and even protagonists as noted in other intestinal diseases. Further studies are thus required to examine the roles of the different elements involved to enable a better approach to CDI.
